# Impact of time-of-shift on diagnostic service requests in a pediatric emergency department: a retrospective study

**DOI:** 10.1007/s11739-025-03954-y

**Published:** 2025-04-30

**Authors:** Pier Mario Perrone, Lorisa Deda, Anna Comotti, Carlo Agostoni, Silvana Castaldi, Gregorio Paolo Milani

**Affiliations:** 1 Department of Pathophysiology and Transplantation, University of Milan, Milan, Italy; 2 Department of Clinical Sciences and Community Health, University of Milan, Milan, Italy; 3 Pediatric Unit, Foundation IRCCS Ca’ Granda Ospedale Maggiore Policlinico, Milan, Italy; 4 Occupational Health Unit, Foundation IRCCS Ca’ Granda Ospedale Maggiore Policlinico, Milan, Italy; 5 Department of Biomedical Sciences for Health, University of Milan, Milan, Italy; 6 Quality Unit, Foundation IRCCS Ca’ Granda Ospedale Maggiore Policlinico, Milan, Italy

**Keywords:** Pediatric emergency department, Shift work, Diagnostic request, Decision fatigue

## Abstract

The demand for medical services and its burden on the healthcare system is worldwide increasing. Factors influencing service requests are still partially unknown. Extended shifts may impair decision-making, potentially affecting the request for ancillary diagnostic procedures. This study aimed to investigate the association between the time-of-shift and the rate of diagnostic service requests in pediatric emergency settings. This single-center observational study was conducted at the pediatric emergency department of the Ca’ Granda Ospedale Maggiore Policlinico in Milan, Italy. The study included patient visits on weekends and public holidays. Data on blood tests, specialist consultations, and imaging requests were extracted. The shift was divided into the first 8 h and the last 4 h, and diagnostic service requests were analyzed using mixed-effects logistic regression models, adjusting for patient urgency and number of patients per shift. A total of 5370 visits were analyzed. At least one ancillary diagnostic procedure was requested in 31% of the visits. There was a 14% higher probability (*p* = 0.04) of requiring ancillary diagnostic procedures during the last 4 h of shifts compared to the first 8 h. This probability increased to 20% (*p* = 0.02) considering exclusively the dayshift. These findings suggest a potential role of shift duration on diagnostic service requests, warranting further multicenter studies to explore this association across various healthcare settings.

## Introduction

The increasing demands on healthcare systems, particularly within emergency departments (EDs), underscore the need to understand factors influencing clinical decision-making and service utilization [1–3]. Additionally, despite rising costs and usage of EDs in many countries, the lack of proportional increases in healthcare economic investments necessitates judicious use of available resources by health professionals [4, 5].

Emergency departments typically operate on a shift system, with day and night shifts mainly lasting between 6 and 12 h. It is known that longer shifts might be associated with a decrease in mental health status among workers [6, 7]. In turn, impaired cognitive functions may be associated with an increased request of unnecessary ancillary examinations such as imaging procedures [8]. Despite the recognition of these issues, it is unknown whether the time-of-shift is associated with different service utilization by physicians working in emergency settings. Therefore, the aim of this study was to analyze the rate of diagnostic service requests in the pediatric emergency department (PED) and its association with the time of the shift.

## Methods

This retrospective single-center observational study was conducted at the Pediatric Emergency Department (PED) of the Fondazione IRCCS Ca’ Granda Ospedale Maggiore Policlinico, Milan, Italy. The study covered the period between January 1st, 2022, and December 31st, 2022. In this hospital, blood laboratory tests, specialist consultations, and instrumental examinations (X-ray and CT scans) are available 24 h a day, every day. On the other hand, primary care pediatricians are only available on weekdays, at various times, to visit children outside the hospital. Therefore, to avoid any potential selection bias due to the different availability of primary care pediatricians, only visits occurring on Saturdays, Sundays, and public holidays were included in this study. At the study site, there are only two shifts on Saturdays, Sundays, and public holidays: a day shift from 8:30 a.m. to 8:30 p.m. and a night shift from 8 p.m. to 8 a.m. During these shifts, the staff consists of one emergency pediatrician, two pediatric residents, two nurses, and one social and health worker.

Data on the following services were collected: blood laboratory tests, specialist consultations, and instrumental examinations (X-ray and CT scans). These ancillary diagnostic procedures are requested through the department’s online system, making it possible to determine the time of each request. Additional examinations (e.g. ultrasounds) were excluded because they are not always available during the 24 h. Data on the urgency code (white, green, yellow and red) assessed during triage and the physician on duty (indicated by an anonymous code) during the shift were also collected. All data were collected by an automated extraction program to avoid reporting errors or missing data. Visits and physicians on duty were collected using anonymized codes.

Since decision making is typically constant over an 8-h shift [9], work shifts were divided into two major groups, corresponding to the first 8 h and the last 4 h of the shift. For each patient, we considered three binary variables: request for i. blood laboratory tests; ii. X-ray/CT scans; and iii. specialist consultations. Additionally, we assessed a variable that scored 1 if at least one of these procedures was requested. These variables were summarized using frequencies and percentages.

The Chi-square test was used to evaluate differences in diagnostic service requests during the two shift periods. Subsequently, mixed-effects logistic regression models were employed to investigate the probability of requesting ancillary diagnostic procedures in the last 4 h of the shift (independent variable), adjusted for the total number of patients during the whole shift and urgency code of the patient. The physician on duty was considered as random effect. Results were presented using odds ratios and their respective 95% confidence intervals. A two-tailed *p* value < 0.05 was considered significant. R software was used for the analyses.

## Results

**Fig. 1:**
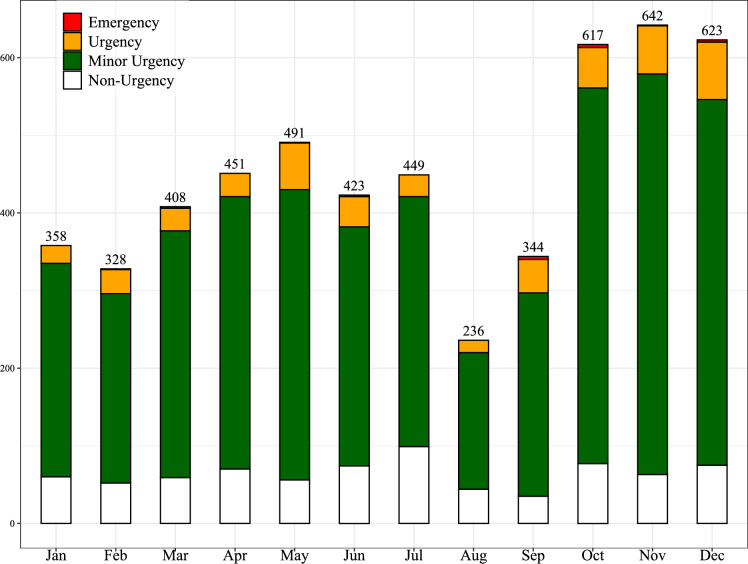
Total number of pediatric emergency department visits: absolute frequencies by month and triage urgency code

A total of 5370 PED visits were included. Figure 1 displays the frequency of visits by month and triage urgency code. No ancillary diagnostic procedure was requested for 69% (*N* = 3691) of the patients, one for 21% (*N* = 1113) and two or more for 10% (*N* = 566). Children with a specialist consultation were 18% (*N* = 980), with blood tests 17% (*N* = 825), and with imaging 9% (*N* = 510). Table 1 reports the type and number of ancillary diagnostic procedures.

**Table 1:** Type and frequencies of ancillary diagnostic procedures

Ancillary diagnostic procedures, *N* (%)	
Specialist consultations	980 (41%)
Blood tests	925 (38%)
X-ray	447 (19.5%)
CT scans	63 (2.5%)

Percentages are calculated based on the total number of procedures (*N* = 2415), which may exceed the number of patients (accesses) undergoing procedures (*N* = 1679)

The requests performed during the first 8 h and the last 4 h of the shift are detailed in Table 2. Considering the whole day, there was an increase of 2.5% in the last 4 h of the shifts compared to the first 8 h. However, this finding did not reach statistical significance. A significant increase of 3.5% between the first 8 compared to the last 4 h was found considering exclusively the dayshift (*p* = 0.006). After adjusting for confounders, the regression model showed a 14% increase in the probability of diagnostic service requests during the last 4 h (OR = 1.14, 95% CI = 1.00–1.30, *p* = 0.04) compared to the first 8 h of the shift considering the whole day, whereas a 20% increase considering exclusively the dayshift (OR = 1.20, 95% CI = 1.03–1.41, *p* = 0.02).

**Table 2:** Total number of accesses, with and without ancillary diagnostic procedures, during the first 8 h and the last 4 h of the shift, along with logistic regression results assessing the likelihood of requiring an ancillary diagnostic procedure in the last 4 h compared to the first 8 h

Ancillary diagnostic procedure’s requests	First 8 h	Last 4 h	Total	*P* value	OR (95% CI)
All working day	3887	1483	5370		
No	2697 (69%)	994 (67%)	3691 (69%)	0.10	
At least one	1190 (31%)	489 (33%)	1679 (31%)		1.14 (1.00, 1.30)
Day shift	1824	1231	3055		
No	1274 (70%)	801 (65%)	2075 (68%)	0.006	
At least one	550 (30%)	430 (35%)	980 (32%)		1.20 (1.03, 1.41)

Data are presented as frequencies and percentages, ORs, and 95% confidence intervals. *P* values were calculated using the Chi-square test

## Discussion

This study shows for the first time that the last 4 h of a 12-h shift were associated with an increase in diagnostic service requests within a pediatric emergency department.

In recent years, there has been a growing attention to the relationship between healthcare organization and clinical practice. This interest is reflected in numerous studies showing the high burden of examination requests on healthcare expenditure [10, 11].

It is well established that the complexity of a disease, its stage, and the presence of comorbidities are associated with an increased demand for diagnostic procedures [12, 13]. This study shed light on the possible effect of ED’s shift duration on the request of ancillary diagnostic procedures in an emergency department setting. The last 4 h of the shift were associated with an increase > 10% in such requests. Although we did not evaluate the appropriateness of the requests, we speculate that the increase in ancillary diagnostic procedures’ requests during the last 4 h of the shift might be due to different possible factors. The high workload can lead to a reduced capacity for discernment or decision-making ability, prompting the overuse of diagnostic instruments to overcome this difficulty [14, 15]. It might be argued that the decision fatigue was due to the high number of patients during the shift more than on the time of shift. However, the probability of requiring an ancillary diagnostic procedure was adjusted for the number of patients cared for during the shift. On the other hand, decision fatigue might also increase the risk of defensive medicine [16]. This approach, which has been reported in pediatrics over several countries, implies that clinical decisions are influenced more by the fear of legal repercussions than by choosing the most appropriate patient management [17]. This attitude may result in an excessive number of ancillary diagnostic procedures’’ requests with potentially negative effects on patients and society [18]. The persistent association between the last 4 h of the shift and the increase of diagnostic service requests after adjusting for the code of triage admission (a proxy a potential disease evolution) suggests the disease severity does not explain this association.

The study showed a wide distribution of visits during the year showing peaks in the autumn and late spring months, which may be attributed to the flare-up of infectious and allergic diseases. Considering that the ED’s overcrowding has been documented in several studies [19], future investigations should assess whether the duration of the shift is differently associated to the request of ancillary diagnostic procedures in the different seasons of the year.

Despite the potential relevance of the findings of the study, some limitations should be recognized. As previously mentioned, we did not evaluate the appropriateness of the ancillary diagnostic procedure’s request. Second, we did not perform any economic analysis. Third, although we speculated that decision fatigue played a relevant role during the last 4 h of the duty, given the retrospective nature of the study we could not directly measure it. Fourth, the study was monocentric, limited to a pediatric center and we could not assess whether a shorter duration of the shift could be associated with a lower request for ancillary diagnostic procedures during the last hours.

In conclusion, this study shows that the last 4 h of a 12-h duty were associated with a relevant increase in ancillary diagnostic procedures’’ request in a pediatric emergency department. Future multicenter studies including both pediatric and adult departments are needed to investigate the interplay among shift duration, decision fatigue and procedures’ request in emergency departments.

## Data Availability

The datasets analyzed during the current study are available from the corresponding author on reasonable request.
